# PM_2.5_ and ozone health impacts and disparities in New York City: sensitivity to spatial and temporal resolution

**DOI:** 10.1007/s11869-012-0185-4

**Published:** 2012-10-12

**Authors:** Iyad Kheirbek, Katherine Wheeler, Sarah Walters, Daniel Kass, Thomas Matte

**Affiliations:** New York City Department of Health and Mental Hygiene, New York, USA

**Keywords:** PM_2.5_, Ozone, Public health burden, Health impact analysis, Vulnerability, Air quality management

## Abstract

Air quality health impact assessment (HIA) synthesizes information about air pollution exposures, health effects, and population vulnerability for regulatory decision-making and public engagement. HIAs often use annual average county or regional data to estimate health outcome incidence rates that vary substantially by season and at the subcounty level. Using New York City as an example, we assessed the sensitivity of estimated citywide morbidity and mortality attributable to ambient fine particulate matter (PM_2.5_) and ozone to the geographic (county vs. neighborhood) and temporal (seasonal vs. annual average) resolution of health incidence data. We also used the neighborhood-level analysis to assess variation in estimated air pollution impacts by neighborhood poverty concentration. Estimated citywide health impacts attributable to PM_2.5_ and ozone were relatively insensitive to the geographic resolution of health incidence data. However, the neighborhood-level analysis demonstrated increasing impacts with greater neighborhood poverty levels, particularly for PM_2.5_-attributable asthma emergency department visits, which were 4.5 times greater in high compared to low-poverty neighborhoods. PM_2.5_-attributable health impacts were similar using seasonal and annual average incidence rates. Citywide ozone-attributable asthma morbidity was estimated to be 15 % lower when calculated from seasonal, compared to annual average incidence rates, as asthma morbidity rates are lower during the summer ozone season than the annual average rate. Within the ozone season, 57 % of estimated ozone-attributable emergency department for asthma in children occurred in the April–June period when average baseline incidence rates are higher than in the July–September period when ozone concentrations are higher. These analyses underscore the importance of utilizing spatially and temporally resolved data in local air quality impact assessments to characterize the overall city burden and identify areas of high vulnerability.

## Introduction

Fine particulate matter (PM_2.5_) and ozone (O_3_) are common combustion-related pollutants that contribute to increased emergency department visits, hospitalizations, and deaths due to respiratory and cardiovascular disease (US EPA 2006a, 2009). While these effects are documented in a large number of peer-reviewed published studies, it is challenging to distill this extensive evidence to describe the public health burden attributable to air pollution to convey the importance of emissions reductions initiatives to elected officials and the general public.

Air quality health impact assessments (HIA) are one approach to synthesizing information about air pollution exposures, health effects, and population vulnerability. Analysis methods have become relatively standardized and used by the US Environmental Protection Agency (US EPA 2008, 2010a) and state and regional groups (NESCAUM 2008) to estimate potential health benefits of air quality regulations. These methods have also been applied in estimating the global and national public health burden of pollutant exposures attributable to current ambient concentrations relative to some estimate of background concentration (Cohen et al. 2005; US EPA 2010a; Fann et al. 2011a, b).

In recent years, there has been growing recognition of the potential value for stakeholder engagement of applying national regulatory HIA methods at a local scale by using health and pollutant exposure data that are more spatially resolved than the county or 12-km model grid cell level typically available and used in air pollution HIA (US EPA 2008, 2010a; Fann et al. 2011a, b; Hubbell et al. 2005; Matte et al. 2009). The use of more finely spatially resolved exposure and health data is essential to support transition from the currently mandated monitor-based National Ambient Air Quality Standards (NAAQS) attainment approach to multipollutant, risk-based air quality management approaches (National Research Council 2004; Dominici et al. 2010). Furthermore, accounting for neighborhood level variation in exposures and morbidity and mortality rates allows for assessment of disparities in air pollution impacts. However, significant methodological challenges need to be addressed when conducting health impact assessments at a local scale, and approaches have been proposed for concentration–response (C–R) function selection, exposure estimation, and baseline incidence data choices (Hubbell et al. 2009; Fuentes 2009).

Seasonal variation in both air pollutants and rates of morbidity and mortality also complicate health impact assessment. For example, asthma hospitalization rates in children typically increase in the early fall and remain higher than summertime rates through much of the spring (Silverman et al. 2005). However, some prior health impact assessments have applied annual average baseline mortality and morbidity rates in calculating health impact estimates associated with changes in air quality that vary by season (Hubbell et al. 2005; US EPA 2008). Recent urban analyses of PM_2.5_ risk applied seasonal baseline morbidity rates when seasonal effect estimates were available (US EPA 2010a). To our knowledge, however, there has not been an analyses examining how sensitive the estimates of the health impacts of changes in air quality are to accounting for seasonal variability in baseline incidence rates.

Using methods we previously employed to derive estimates of the public health burden attributable to current levels of PM_2.5_ and O_3_ in New York City (NYCDOHMH 2011a), we assessed how estimates vary with differing spatial resolution (county vs. neighborhood level) of the analysis and the temporal resolution (annual average vs. seasonal) of baseline health incidence rates. We also quantified disparities in impacts by area-based poverty concentration.

## Methods

### Overall approach

We calculated the burden of exposure to current levels of ambient PM_2.5_ and ozone in New York City using previously described methods (Hubbell et al. 2009; Fann et al. 2011a, b). Briefly, we applied evidence from published time-series and cohort studies relating ambient air pollutant concentrations to health outcomes to local data on air pollutant levels, baseline mortality and morbidity rates, and exposed populations. Changes in morbidity and mortality attributed to changes in air pollution were calculated using health impact functions derived from log-linear models relating the risk of disease or death to ambient concentrations of air pollutants of interest:

$$ \varDelta I = \left( {1 - {e^{{ - \beta \varDelta X}}}} \right) \times P \times {I_0} $$

Where Δ*I* is the change in the number of health events associated with the change in air pollutant concentration (Δ*X*), *β* is the effect coefficient from the epidemiological study, *P* is the exposed population, and *I*_0_ is the baseline rate of disease or death.

All health impact calculations were conducted using US Environmental Protection Agency’s Benefits Mapping and Analysis Program (BenMAP) Version 4.0, a GIS-based platform that allows analysts to estimate the health impacts associated with user-defined changes in air quality (US EPA 2010b). BenMAP has been used extensively for regulatory applications such as Environmental Protection Agency (EPA)’s analysis of the Federal Transport Rule (US EPA 2011), in evaluation of ozone and PM_2.5_ National Ambient Air Quality Standards (US EPA 2006b, 2008), and as part of State air quality management planning (NYSDEC 2011).

### Current air quality data

Air quality data from EPA’s Air Quality System (AQS) were acquired from all regulatory monitors in the five counties of New York City and the seven adjacent counties in New York State and New Jersey for the 3 years from 2005 to 2007.

PM_2.5_ data were obtained from 24 monitors collecting integrated 24-h filter-based samples by federal reference methods. Three monitors reported data on a daily schedule while 18 reported on an every third day schedule and three reported on an every sixth day schedule. Daily average values at each monitor were averaged by quarter (Jan 1–March 31, April 1–June 30, July 1–Sep 30, and Oct 1–Dec 31) within each year, and then each quarter was averaged across 3 years. These 3-year quarterly averages were used to characterize baseline air quality while reducing the influence of year-to-year variation due to weather.

Ozone data were obtained for the seven monitors in the region reporting data from 2005 to 2007. Hourly ozone data were used to calculate daily exposure metrics including the daily 8-h maximum, 24-h average, and 4-h afternoon average (1:00–5:00 p.m.). Daily metrics at each monitor were then averaged for each of the two quarters comprising the New York City ozone season (April 1–June 30, July 1–Sep 30) within each year, and then each quarter was averaged across 3 years.

Average concentrations for each quarter were assigned to each of 42 zip code aggregate-based New York City United Hospital Fund (UHF) neighborhoods using an averaging approach within BenMAP known as the Voronoi neighbor averaging (VNA; US EPA 2010b). In short, the VNA algorithm, used in prior air quality HIAs (Hubbell et al. 2005; Fann and Risley 2011), identifies monitors that best surround a point of interest (in this case, the centroid of a given neighborhood or county) then calculates the inverse distance-weighted average concentration of the values from these monitors.

### Comparison scenario

We estimated the burden of exposures to current levels of PM_2.5_ and ozone based on the difference relative to non-anthropogenic, policy-relevant background concentrations (PRB). These background concentrations are derived through atmospheric modeling where all man-made emissions have been removed from the model. For PM_2.5_, we applied the northeast, season specific, PM_2.5_ PRB concentrations published in EPA’s 2009 Integrated Science Assessment for Particulate Matter (US EPA 2009) based on modeling performed with the Community Multi-Scale Air Quality Modeling System and the Goddard Earth Observing System (GEOS)-Chem model. Policy-relevant background ranged from 0.67 to 0.87 μg/m^3^ depending on season, or approximately 5 % of current average PM_2.5_ concentrations in New York City.

For ozone, we applied PRB estimates modeled by Fiore et al. (2004) using the GEOS-Chem model. In their analysis, Fiore et al. (2004) reported 4-h, afternoon average background ozone estimates during the April to October ozone season for four regions of the USA. We converted the 4-h, afternoon average PRB estimate in the Northeast to a 8-h maximum and 24-h average PRB by computing the ratio of the 4-h average to the 8-h maximum or the 24-h average, calculated from the hourly monitoring data from the sites and time period used in our analysis (Anderson and Bell 2010). Policy-relevant background concentrations were estimated at 21.2 and 20.0 ppb for the 8-h maximum in April–June and July–September, respectively, or approximately 45 % of current average ozone concentrations in New York City and a smaller proportion of the concentration on days with poor air quality.

### Selection of concentration–response functions

We reviewed recent epidemiological studies of the relationship of PM_2.5_ and O_3_ to mortality, hospital admissions, and emergency department visits and identified those we judged most relevant to the current New York City population for use in the main analyses (Tables 1 and 2). All studies were published in peer-reviewed scientific journals and studies of New York City were used when possible. If local studies were not available, we used recent large, multicity studies or those included in EPA risk analyses (US EPA 2008, 2010a).

**Table 1:** Effect estimates used in PM_2.5_ health burden analysis

Health effect	Outcome definitions	Age group	Exposure metric	Effect estimate	Study location	Source of effect estimate
Premature mortality	All ICD10-coded underlying causes of death	30 and above	Annual average	Relative risk of 1.056 per 10 μg/m^3^ increase in PM_2.5_, model adjusted for seven ecological covariates.	USA, 116 cities	Krewski et al. (2009)
Emergency department visits—asthma	ICD-9:493	All ages	Daily 24-h average	Relative risk of 1.23 (warm season) and 1.04 (cold season) per 25.4 and 21.7 μg/m^3^ respective increase in PM_2.5_	New York City	Ito et al. (2007)
Hospital admissions—all cardiovascular causes	Outcomes listed as “emergency” or “urgent” as the sum of ICD-9: 402, 410, 414, 427, 428, and 430	40 and above	Daily 24-h average	0.8 % (Warm season) and 1.1 % (cold season) increase in daily cardiovascular disease hospitalizations per 10 μg/m^3^ increase in PM_2.5_	New York City	Ito et al. (2010)
Hospital admissions—all respiratory causes	ICD-9: 490-448	20–64 Years	Daily 24-h average	2.2 % Increase in daily chronic respiratory disease hospitalizations per 10 μg/m^3^ increase in PM_2.5_	Los Angeles, CA	Moolgavkar (2000)
	ICD-9: 460–519 (cases admitted from the emergency room)	65 and above	Daily 24-h average	1.79 % (Winter), 4.34 % (spring), 1.26 % (summer), 1.52 % (autumn) increase in respiratory disease hospitalizations per 10 μg/m^3^ increase in PM_2.5_	26 US communities	Zanobetti et al. (2009)

**Table 2:** Effect estimates used in ozone health burden analysis

Health effect	Outcome definitions	Age group	Exposure metric	Effect estimate	Study location	Source of effect estimate
Premature mortality	Cause specific mortality ICD-9: 390–448, 490–496, 487, 480–486, 507	All ages	Daily 24-h average	2.33 % Increase in cardiovascular and respiratory mortality per 10 ppb increase in ozone levels over the previous week	New York City	Huang et al. (2005)
Emergency department visits—asthma	ICD-9:493	All ages	Daily 8-h maximum	Relative risk of 1.32 per 53.5 ppb increase in ozone	New York City	Ito et al. (2007)
Hospital admissions—asthma	ICD-9:493, cases listed as “emergency” or “urgent”	All ages	Daily 8-h maximum	Relative risk of 1.06–1.20 (varies by age group) per 22 ppb increase in ozone	New York City	Silverman and Ito (2010)

### Baseline incidence and population data

Mortality data for New York City residents were provided by the New York City Health Department’s Bureau of Vital Statistics for 2005 through 2007. Based on the underlying cause of death, daily counts were summarized and rates of all-cause, cardiovascular, and respiratory mortality were calculated across 22 age and gender groupings (44 total) for each county and 42 UHF neighborhoods. Hospital admissions and emergency room visits data for New York City residents were obtained from the New York Statewide Planning and Research Cooperative System for the same 3-year period (2005–2007). Using diagnostic codes in the hospital discharge data, case definitions were matched to the case definitions used in each of the concentration–response functions used in this analysis. For all mortality and morbidity data, we calculated quarterly rates that matched the quarter definitions in the air quality data and annual average rates, at the UHF and county-level spatial scale. Rates were then averaged over the 3-year period to reduce the influence of random year-to-year variation in rates and to match air quality data.

The 44 age- and sex-specific population estimates for 2005 through 2007 were produced by the New York City Department of Health and Mental Hygiene based on the US Census Bureau Population Estimate Program, supplemented by housing data obtained from the New York City Department of City Planning (NYCDOHMH DES).

To develop estimates of disparity by neighborhood socio-economic status, we stratified the 42 UHF neighborhoods into three poverty tertiles, defined by the percent of neighborhood residents at less than 200 % of the federal poverty threshold, based on data from the 2000 US Census.

### Sensitivity analysis

We repeated the citywide pollutant-attributable health burden calculations with varying air quality and baseline health input data to estimate the sensitivity of the final results to method choices. These analyses included:

### Sensitivity to spatial scale

We calculated the citywide impact associated with the difference in average air quality levels between the policy relevant background and the 2005–2007 concentrations at the UHF neighborhood level and at the county level. In each case, we matched the baseline incidence rates to the spatial scale of the air quality exposure estimates and summed sub-area impacts to compute estimates of the total citywide burden.

To examine within-city variation in exposure and susceptibility, we computed correlations among air pollutant concentrations, incidence counts, and poverty at the neighborhood level. To assess disparities in air pollution health impacts, UHF neighborhood-level estimates were then aggregated to three neighborhood poverty categories, based on the percent of UHF residents living below 200 % of the federal poverty level grouped by neighborhood tertiles. We then compared the intracity impact gradients and disparities from the neighborhood- and county-scale analyses. For the latter, we estimated neighborhood-level impacts by applying county-level incidence rates and air pollution estimates to all neighborhoods within a county. Gradients were compared using ranges and rank correlations among impact rates computed by the two methods. We also compared the ratios of impact rates in high- compared to low-poverty neighborhoods computed by each method.

### Sensitivity to temporal resolution of incidence data

We first used quarterly baseline incidence rates and air quality data to calculate the citywide burden by quarter then summed the results to produce estimates of attributable health events per year. These estimates were compared to those based on annual average health outcome rates and seasonal air quality data.

## Results

### Air quality and incidence rate estimates

Both PM_2.5_ and ozone varied seasonally, with PM_2.5_ levels showing peak values in third (summer) quarter when levels were 17 % higher than the annual average and showing the lowest values in the second (spring) quarter when values were 10 % lower than the annual average (Table 3). Ozone levels also peaked in the third quarter, showing 8 % higher 8-h maximum concentrations than the second quarter.

**Table 3:** Average concentrations of PM_2.5_ and ozone in 2005–2007 and natural background levels used for comparison scenario

Pollutant	Quarter	Averaging time	2005–2007 NYC average concentration		Policy relevant background
			Area-wide monitor mean	UHF neighborhood range (*N* = 42)	
PM_2.5_ (μg/m^3^)	Jan–Mar	24-h average	14.1	11.6–17.1	0.85
	Apr–Jun		12.6	10.2–14.0	0.78
	Jul–Sep		16.4	13.8–17.0	0.67
	Oct–Dec		12.7	10.7–13.9	0.68
O_3_ (ppb)	Apr–Jun	8-h Max	43.8	36.3–45.0	21.20
		24-h Mean	30.3	23.8–31.5	14.70
	Jul–Sept	8-h Max	47.2	40.5–49.0	20.00
		24-h Mean	30.2	24.6–32.0	13.00

**Fig. 1:**
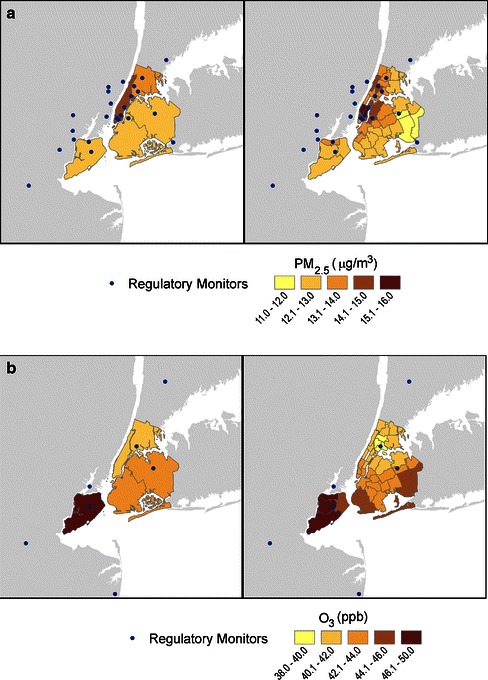
2005–2007 Annual concentrations of PM_2.5_ and ozone, inverse distance weighted average across county and UHF neighborhood (VNA method). **a** PM_2.5_, annual average of daily mean; **b** ozone, ozone-season average of daily 8-h maximum

Interpolation of monitor data to the UHF level provided increased spatial variability in air pollutant concentrations than the county-level interpolation (Fig. 1). For county-level estimates, the ranges of the average concentrations were 17 and 29 % of the mean for PM_2.5_ and ozone, respectively. The corresponding ranges of the UHF neighborhood level estimated concentrations were 30 and 39 % of the mean concentrations, respectively.

**Fig. 2:**
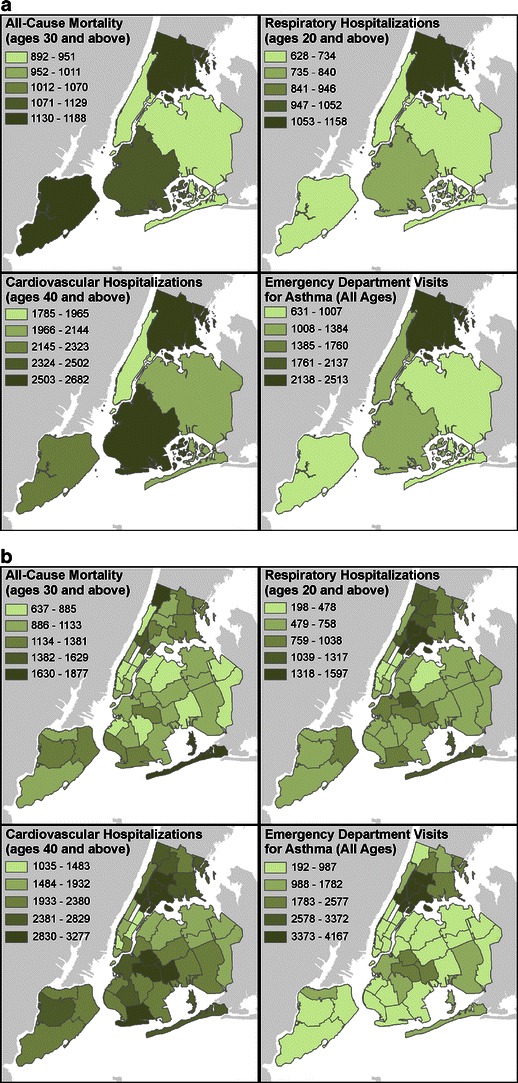
Distribution of baseline mortality and morbidity across NYC counties and neighborhoods (annual rate per 100,000 persons, 2005–2007 average); **a** county, **b** UHF

In New York City, there is substantially more spatial variability at the neighborhood than at the county level for baseline incidence rates of all-cause mortality, hospitalizations for cardiovascular and respiratory disease, and emergency department visits for asthma (Fig. 2). Greater neighborhood variability is especially notable for asthma-related emergency department visits where county-level rates vary four-fold, with highest rate in the Bronx, while UHF level rates vary 20-fold with the highest rates found in the neighborhoods of Northern Manhattan followed by the South Bronx. Similarly, hospitalizations for respiratory causes vary by eight-fold across UHF neighborhood, with highest rates in the Bronx and Northern Manhattan while rates only vary by 1.8-fold at the county level. Relatively less spatial variability is seen in mortality and cardiovascular hospitalizations with threefold and 3.2-fold differences across UHF neighborhoods, respectively.

Estimated neighborhood level PM_2.5_ exposure levels were generally not significantly associated with baseline counts of health events while ozone exposure levels were found to be significantly negatively associated with asthma emergency department visits and hospitalizations (Table 4). Neighborhood poverty was not associated with PM_2.5_ levels but significantly negatively associated with ozone levels. Conversely, neighborhood poverty level was found to be significantly positively correlated with all health endpoints except mortality.

**Table 4:** Spearman correlations (rho) among neighborhood exposure and susceptibility variables

		Jan–Mar		Apr–Jun		Jul–Sep		Oct–Dec	
		Pollutant Concentration	Poverty	Pollutant Concentration	Poverty	Pollutant Concentration	Poverty	Pollutant Concentration	Poverty
PM_2.5_									
Mortality	Ages 30 and above	−0.16	0.12	−0.2	0.11	−0.14	0.13	−0.06	0.12
Emergency department visits for Asthma	All ages	0.06	0.74*	−0.02	0.72*	−0.13	0.73*	0.11	0.75*
Hospital admissions, respiratory causes	Ages 20 and above	0.02	0.32*	0.06	0.56*	−0.21	0.55*	0.05	0.59*
Hospital admissions, cardiovascular causes	Ages 40 and above	−0.28	0.56*	−0.2	0.35*	−0.32*	0.35*	−0.19	0.33*
Neighborhood poverty		0.11	1	0.25	1	0.02	1	0.23	1
Ozone									
Mortality	All ages			0.13	−0.02	0.12	−0.01		
Emergency department visits for asthma	All ages			−0.43*	0.72*	−0.42*	0.73*		
Hospital admissions, asthma	All ages			−0.38*	0.74*	−0.41*	0.75*		
Neighborhood Poverty				−0.34*	1	−0.31*	1		

*p < 0.05, significant

### Sensitivity of overall impact estimates to spatial scale

Citywide health impacts were found to be insensitive to the geographic level used to average air quality and health outcome incidence data. Differences between estimates based on county level compared to neighborhood level analyses were in all cases less than 1 % (Table 5)

**Table 5:** Comparison of estimated citywide annual burden of PM_2.5_ and ozone using county and neighborhood level air quality and incidence data

Pollutant	Health effect	Age group	County level analysis		UHF level analysis	
			Citywide count (95 % CI)	Citywide rate per 100,000 (95 % CI)	Citywide count (95 % CI)	Citywide rate per 100,000 (95 % CI)
PM_2.5_	Premature mortality	30 and above	3,190 (2,210, 4,140)	65 (45, 85)	3,180 (2,200, 4,130)	65 (45, 84)
	Hospital admissions—cardiovascular conditions	40 and above	930 (210, 1,630)	26 (5.8, 45)	920 (210, 1,630)	26 (5.9, 46)
	Hospital admissions—respiratory conditions	20 and above	1,200 (460, 1,930)	20 (7.6, 32)	1,200 (460, 1,930)	20 (7.6, 32)
	Emergency department visits—asthma	All ages	5,970 (3,630, 8,250)	73 (44, 100)	6,000 (3,650, 8,300)	73 (44, 100)
O_3_	Premature mortality	All ages	400 (200, 590)	4.9 (2.5, 7.2)	400 (200, 600)	4.9 (2.5, 7.3)
	Hospital admissions—urgent asthma	All ages	870 (500, 1,230)	11 (6.1, 15)	870 (500, 1,220)	11 (6.0, 15)
	Emergency department visits—asthma	All ages	4,660 (3,380, 5,910)	57 (41, 72)	4,610 (3,340, 5,840)	56 (41, 71)

### Neighborhood disparity by poverty status

**Fig. 3:**
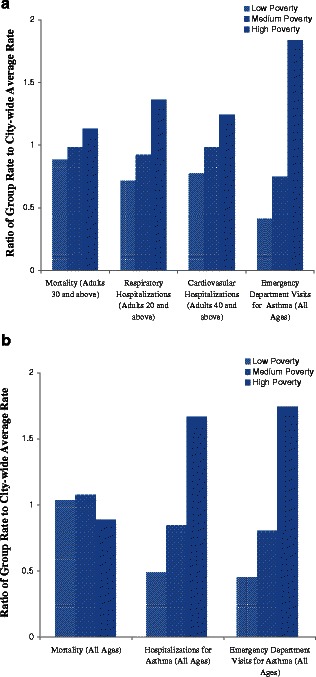
PM_2.5_ and ozone-attributable health burden by neighborhood poverty status; **a** PM_2.5_, **b** ozone

For all endpoints except ozone-attributable mortality, higher rates of air pollutant-attributable events are associated with neighborhood poverty (Fig. 3). The largest disparities are estimated for PM_2.5_ and ozone-attributable asthma emergency department visit rates, which are 4.5 and 3.9 times higher, respectively, in high-poverty neighborhoods, compared to low-poverty neighborhoods. While rates of PM_2.5_-attributable deaths were 28 % higher in high- compared to low-poverty neighborhoods, the rates of O_3_-attributable mortality rates were relatively evenly distributed by neighborhood poverty.

The neighborhood-level analysis revealed important subcounty variability in pollutant-attributable health impacts not observed in the county-level analysis. For example, the highest and lowest citywide asthma rates are both found in neighborhoods within New York County (Manhattan): Central Harlem and Greenwich Village/SoHo, respectively. These neighborhoods also fall in the extremes of poverty concentration, with Central Harlem in the high-poverty category and Greenwich Village/SoHo in the low-poverty category.

### Sensitivity of the gradient in impact to spatial scale

The range in estimated rates of air pollution attributable events was approximately two to three times greater when applying UHF-level rates compared to applying county-level rates; the neighborhood rankings of attributable impact rates using the two methods were only moderately correlated (Spearman’s rho, 0.41–0.60; Table 6). Comparisons of relative burden in impacts by neighborhood poverty status showed wider disparities when the analysis was conducted using UHF-specific rates as opposed to county-specific rates for all endpoints except ozone-attributable mortality, where limited disparity was observed in either method.

**Table 6:** Comparison in neighborhood level ranges of relative pollutant-attributable burdens and disparity by poverty status based on analysis spatial scale

		Relative burden (rate per 100,000 persons)	
		Range in neighborhood estimates	Ratio of rates in high- to low-poverty neighborhoods
PM_2.5_-attributable mortality (above 30)	UHF analysis	75	1.28
	County analysis	25	1.14
	Spearman’s correlation	0.55	
PM_2.5_-attributable cardiovascular hospitalizations (Above 40)	UHF analysis	39	1.60
	County analysis	16	1.23
	Spearman’s correlation	0.53	
PM_2.5_-attributable respiratory hospitalizations (above 20)	UHF analysis	26	1.90
	County analysis	11	1.31
	Spearman’s correlation	0.60	
PM_2.5_-attributable emergency department visits for asthma (above 30)	UHF analysis	250	4.46
	County analysis	110	1.61
	Spearman’s correlation	0.46	
O_3_-attributable mortality (all ages)	UHF analysis	9	0.86
	County analysis	4	0.96
	Spearman’s correlation	0.47	
O_3_-attributable hospital admissions for asthma (all ages)	UHF analysis	24	3.40
	County analysis	13	1.67
	Spearman’s correlation	0.45	
O_3_-Attributable emergency department visits for asthma (all ages)	UHF analysis	179	3.85
	County analysis	71	1.56
	Spearman’s correlation	0.41	

### Sensitivity to temporal resolution

**Fig. 4:**
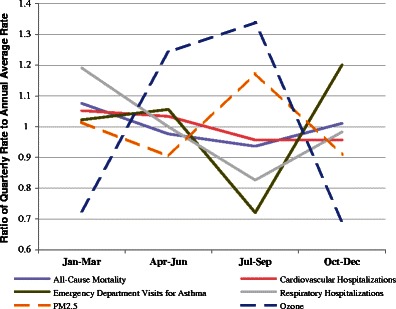
2005–2007 Quarterly average patterns of baseline incidence rates and pollutant concentrations for endpoints considered in health impact assessment

Rates of emergency department visits for asthma and respiratory hospitalizations varied significantly by quarter, with the lowest rates occurring in the months of July–September (27 and 21 %, respectively, below the annual average rates) during the peak ozone season (Fig. 4). Mortality and cardiovascular hospitalization rates had less seasonal variation, but were also lower during the July–September period compared to the annual average (by 6 and 3 %, respectively).

Estimated PM_2.5_-attributable health events based on quarterly incidence rates were generally similar to those based on annual average rates; the largest difference was for cardiovascular hospitalizations which were 7 % lower using seasonal rates (Tables 7 and 8).

**Table 7:** Comparison of estimated citywide annual burden of PM_2.5_ using quarterly and annual average incidence rates

		Attributable counts		Attributable rate per 100,000 persons	
Health effect	Risk estimate	Annual estimate derived from quarterly rates (95 % CI)	Annual estimate derived from annual incidence rate (95 % CI)	Annual estimate derived from quarterly rates (95 % CI)	Annual estimate derived from annual incidence rate (95 % CI)
Mortality (above 30)	Annual risk estimate in both cases	3,180 (2,200, 4,130)	3,230 (2,240, 4,200)	64 (45, 83)	66 (44, 86)
Hospital admissions—all cardiovascular causes (above 40)	Seasonal risk estimates for quarterly rates, year-round risk estimates for annual rates	920 (210, 1,630)	990 (490, 1,500)	25 (5.8, 45)	28 (14, 41)
Hospital admissions—all respiratory causes (above 20)		1,200 (460, 1,930)	1,190 (690, 1,670)	20 (7.5, 31)	20 (11, 28)
Emergency department visits for asthma (all ages)		6,000 (3,650, 8,300)	5,700 (4,000,7,390)	72 (44, 100)	69 (48, 90)

**Table 8:** Comparison of estimated citywide annual burden of ozone using quarterly and annual average incidence rates

		Attributable counts		Attributable rate per 100,000 persons	
Health effect	Risk estimate	Ozone season estimate derived from quarterly rates	Ozone season estimate derived from annual incidence rate	Annual estimate derived from quarterly rates (95 % CI)	Annual estimate derived from annual incidence rate (95 % CI)
Mortality (all ages)	Ozone season	400 (200, 600)	420 (210, 630)	5.1 (2.6, 7.5)	5.2 (2.6, 7.7)
Hospital admissions-asthma (all ages)		870 (500, 1,220)	1,000 (590, 1,450)	11 (6.1, 15)	13 (7.2, 18)
Emergency department visits for asthma (all ages)		4,610 (3,340, 5,840)	5,310 (3,850, 6,730)	57 (41, 72)	65 (47, 82)

In contrast, O_3_-attributable asthma hospitalizations and emergency department visits estimates were more sensitive to the use of seasonal rates; both were 15 % lower than those calculated using the more conventional approach of applying annual average rates. In addition, seasonally stratified analysis revealed that although average 8-h maximum ozone levels were 8 % higher in the July–September period as compared to the April through June period, an estimated 53 % of all ozone-attributable emergency department visits and 57 % of ozone-attributable emergency department visits among children under 18 years of age occurred in the April–June period.

## Discussion

Our analyses found that in New York City, citywide health impact estimates were relatively insensitive to the geographic level (county compared to neighborhood) used to average air quality and health outcome incidence data. However, estimated pollutant-attributable mortality and morbidity varied widely across neighborhoods with differing socio-economic status. Baseline mortality and morbidity rates varied by season, with largest temporal variability found in the rates of emergency department visits for asthma and respiratory hospitalizations. Comparing the use of annual incidence rates versus seasonal rates for a citywide health impact analysis revealed the largest differences in ozone-attributable asthma hospitalizations and emergency department visits because high ozone concentrations occur during times of lower than average baseline incidence rates.

Across all pollutants and health endpoints, we found similar estimates in the burden when calculating at the UHF or county level then aggregating to a citywide estimate. This can be explained by the fact that the change in air quality associated with the rollbacks was relatively poorly correlated geographically with the baseline incidence counts and UHF-level pollutant gradients were not large within individual counties or across the city as a whole. For pollutant/health endpoint combinations where there were limited associations between baseline incidence counts and pollutant levels at the neighborhood (UHF) level, we saw slight differences in the estimates. In other locations where there may be large exposure gradients and strong associations between neighborhoods with high pollution levels and density of susceptible populations, averaging data to a coarser spatial scale for an impact analysis could potentially bias estimates of citywide impacts. Additional factors such as monitor locations and population density will affect exposure assignment and should be considered in developing an appropriate spatial scale for an analysis.

Neighborhood baseline incidence rates serve as one surrogate for relative vulnerability to air pollution health impacts. In New York, a city with very affluent and poor neighborhoods, variation in baseline rates, rather than air quality, account for most of the disparities in pollutant-attributable health where, for example, high-poverty neighborhoods experience 4.5 times higher burden PM_2.5_-attributable asthma department visits as compared to low-poverty neighborhoods. Although not generally available, relative risks may also vary between neighborhoods based on spatial differences in exposure, co-pollutants, and susceptibility including modification of effect by neighborhood traffic density (Ito et al. 2009). Future improvements in air pollution benefits analyses could come from identification of neighborhood modifiers of air pollution C–R functions and improvements in spatial resolution of air quality monitoring data.

Air quality managers relying on health incidence data available within the BenMAP tool or from publicly accessible sources would typically only able to conduct a county-level analysis. Our analysis of the gradients in pollutant-attributable health impacts found that county-level assessments may not reflect the wider range and greater disparity in impact within-city revealed using neighborhood-scale data.

This level of analysis can be particularly important when evaluating the benefit of control strategies where population susceptibility or air quality improvements may be unevenly distributed within an urban area. For example, in estimating the benefits associated with controls on power plants in the Washington DC area, Levy et al. (2002) reported that an impact assessment that stratified baseline mortality rates and PM_2.5_ relative risk by population susceptibility did not result in significantly different citywide mortality benefits as compared to unstratified analysis. However, the model that included stratification by education status highlighted the disproportionate impact on less-educated populations. Multipollutant risk-based strategies being developed by EPA have underscored the importance of fine scale, local data in assessing both the magnitude and distribution of benefits associated with air quality improvement strategies, demonstrating that control strategies focused on maximizing benefits in susceptible populations increased overall benefits by almost two-fold while reducing disparities across the population (Fann et al. 2011b). Fine-scale analyses that best reflect neighborhood health conditions are also appropriate in evaluating local initiatives that are aimed at reducing emissions in neighborhoods with high morbidity, such as efforts in New York City that prioritize cleaner fuel boiler conversions in schools in neighborhoods with high asthma rates (NYC 2011).

While environmental justice concerns have traditionally focused on the gradients in air quality exposure, our findings highlight the importance of including gradients in susceptibility, as reflected by baseline morbidity and mortality rates, among groups of differing socioeconomic status (SES). In this analysis, we found no significant gradient in PM_2.5_ exposures between neighborhoods of differing poverty status, while ozone levels were slightly higher in higher SES communities due to elevated NO_x_ concentrations in areas of Northern Manhattan and the Bronx that increase ozone scavenging (US EPA 2006a). Despite the lack of PM_2.5_ exposure gradients and negative associations between ozone levels and poverty status, we found wide disparities across SES groups in pollutant-attributable health events due to differences in population susceptibility. Prior US national-level analyses have noted that across the country, the percent poverty of a county was positively associated with PM_2.5_ levels while an opposite relationship was observed for ozone, with indication that the relationship between county poverty and air quality can vary by geographic region (Miranda et al. 2011). Similarly, European studies have found that while there are not consistent patterns in exposure gradients between SES groups, individuals of low SES were subject to greater health effects of ambient air pollution (Deguen and Zmirou-Navier 2010).

When varying the averaging time of the baseline incidence rates, we found that differences in PM_2.5_-attributable health impacts were relatively small. The insensitivity of the estimates to temporal resolution of the baseline incidence data is partly due to limited variability in PM_2.5_ levels across seasons and the fact that seasonal patterns in PM_2.5_ and health incidents rates were not strongly associated (either positively or negatively).

Conversely, we found that applying an annual incidence rate likely overestimates O_3_-attributable asthma hospitalizations and emergency department visits and would similarly overestimate the benefits of reducing O_3_ concentrations. This bias can be explained by the opposing patterns of the baseline rates and ozone concentrations, where peak ozone levels in the third quarter correspond to low baseline rates of asthma-related hospitalizations and emergency department visits. Seasonally stratified analyses also indicated that the majority of ozone-attributable emergency department visits in New York City occur in the earlier portion of the ozone season (April–September).

The seasonality of baseline incidence rates should be considered particularly when the impact of control strategies varies by season. For example, regulatory impact analyses for ozone NAAQS that have applied the readily available annual average baseline incidence rates (US EPA 2008) may overestimate asthma-related impacts due to lower summertime asthma incidence as compared to the annual average. Prior evaluations of ozone-season specific emissions trading strategies such as NO_x_ SIP Call (Burtraw et al. 1998; Environmental Protection Agency 1998) included similar limitations. Air quality advisories for ozone, during which sensitive populations are encouraged to limit exposures and all are encouraged to limit driving, tend to occur later in the ozone season, corresponding to peak ozone concentrations. Our findings suggest that more consideration of springtime ozone impacts is needed in developing air quality management and public health protection strategies. Additionally, health impact assessments of measures to reduce emissions from heating fuels such as those developed in New York City through PlaNYC 2030 (NYC 2011) should account for seasonal variation in emissions and health incidence rates.

A significant limitation in this work and any health impact analysis is uncertainty in underlying data and assumptions, many of which are difficult to quantify. By using local data on neighborhood health events, we have improved upon prior work that assume local rates can be approximated using national, regional, or county data. However, the magnitude of our pollutant-attributable estimates is limited by the uncertainty in the risk estimates derived from the epidemiological literature. For the short-term risk estimates, we have attempted to reduce this uncertainty by applying C–R functions from studies conducted on New York City populations where these estimates have been available, presumably better reflecting underlying susceptibility, local air pollutant mixtures, and PM_2.5_ composition. However, for endpoints without published epidemiological studies on local populations, we have relied on effect estimates from studies either conducted in other cities or from larger multicity studies, which may result in additional uncertainty in the pollutant-attributable impact estimates (Hubbell et al. 2009). For example, in our analyses, we calculated PM_2.5_-attributable long-term mortality effects using the Krewski et al. (2009) analysis of the American Cancer Society (ACS). Although this is the largest and most recent study on the effects of PM_2.5_ on mortality, the ACS population has a smaller proportion of low income and minority participants than the New York City population. Our estimate of the PM_2.5_ burden would have been more than twice as large had we applied a concentration–response function based on the Laden et al. (2006) analysis of the more diverse Harvard Six Cities cohort (NYCDOHMH 2011a).

An additional limitation in our analysis is that we have assumed that the same risk estimates apply across all neighborhoods. For the long-term mortality effects examined, the Krewski et al. (2009) study used the citywide average PM_2.5_ concentrations across cities as the exposure contrast (i.e., the subjects in the same city are assigned the same PM_2.5_ level), and thus no within-city exposure variations were considered. Although Krewski et al. (2009) did examine modification by level of education, finding that mortality risk estimates increased with decreasing level of education, the published data do not include sufficient information to derive New York City neighborhood-specific concentration response functions.

Similarly, for the short-term effects studies, sub-urban C–R functions are generally not available for use in health impact assessments. National, multicity studies can examine effect modification by city- or county-level characteristics and provide evidence that socioeconomic status may modify short-term risks of ozone-attributable mortality (Bell and Dominici 2008) but do not quantify how neighborhood level concentration response functions vary across New York City neighborhoods. Within many cities, population sizes at the neighborhood level limit power for time-series or case-crossover analysis, resulting in larger uncertainty in risk estimates. Ongoing work is currently exploring the use of spatially stratified time-series models and other approaches for developing neighborhood level CR function estimates (Ito et al. 2009) that better reflect neighborhood differences in susceptibility to air pollution effects.

Without neighborhood level data on air pollution exposures that corresponded to neighborhood health incidence data, we elected to characterize subcounty exposures to ozone and PM_2.5_ as the inverse distance weighted average of nearby regulatory monitors (EPA VNA methodology). We recognize that regulatory monitoring networks are subject to spatial limitations that may not adequately characterize fine-scale concentration gradients found in urban areas. Ongoing and future work may help reduce spatial uncertainties in exposure, including applying data from high density monitoring networks with land-use regression (LUR) modeling (NYCDOHMH 2011b) or atmospheric modeling results at fine spatial scales (Wesson et al. 2011) that can supplement monitoring data. These methods are subject to their own limitations stemming from emissions inventories, source surrogate data, and fine-scale meteorology data. While beyond the scope of this paper, we have found that LUR-based neighborhood level exposure estimates for PM_2.5_ mass are more variable than the estimates based on inverse distance weighting (IDW) of regulatory monitors used in this paper (coefficient of variation = 0.13 and 0.07 for LUR-based and IDW estimates, respectively; NYC 2012). Future investigations will apply these estimates as multiple years of data become available and models are developed for other pollutants.

Conducting an air quality health burden analysis such as presented here includes the assumption that the same relationship between pollutant concentrations and health risk exists at levels well below the lowest measured levels in the epidemiological literature. While this introduces additional uncertainty in the shape of the dose–response curve at lower levels, available data does not suggest a health effect threshold in the range of concentrations relevant to our analysis. While this paper examined how overall health burden of PM_2.5_ and ozone varied with choices of spatial and temporal resolution, we recognize that our overall estimates of pollution-attributable impacts are sensitive to other method choices, including uncertainties explored elsewhere (Hubbell et al. 2009; Fuentes 2009).

## Conclusions

We evaluated the sensitivity of estimates of citywide PM_2.5_ and ozone attributable health burden in New York City to spatial and temporal scale of baseline air pollution and health incidence data. While aggregated citywide estimates varied little by spatial resolution of air quality and baseline health incidence data, the finely stratified neighborhood-level analysis revealed significant sub-county variability in pollutant-attributable burdens, with significant disparities observed between low- and high-poverty neighborhoods. Comparisons of citywide impact estimates calculated from seasonal vs. annual incidence rates found the largest differences in ozone-attributable hospitalizations and emergency department visits due to simultaneous occurrence of lower-than-average baseline rates and high ozone concentrations, with the majority of ozone-attributable asthma emergency department visits occurring in the first half of the ozone season.

These findings indicate that the choice in methodology should ultimately be guided by the goal of the specific impact analysis. In analyses of broad control strategies that evenly affect urban areas spatially and temporally, county-level analysis may suffice, particularly if the planner is interested in evaluating citywide impacts of less variable health events, as detailed in the analysis of PM_2.5_-attributable mortality in New York City. However, in analyzing local policies that unevenly affect concentrations across neighborhoods within an urban area, our analyses demonstrate the importance of fine spatial resolution baseline incidence data that properly characterize subcounty differences in susceptibility and disparities by socio-economic status. Similarly, for health endpoints that vary temporally, such as in the example of ozone-attributable emergency department visits, using annualized incidence rates can bias citywide health impact estimates. These local scale assessments can help decision-makers target interventions in neighborhoods with relatively higher burdens of deaths and disease attributable to air pollution while providing useful data for community stakeholders in neighborhoods that suffer from relatively higher rates of morbidity than the county or city as a whole. Similarly, applying this level of analysis to future air quality policy development will help prioritize strategies that result in greater health benefits overall and reduce disparities in impacts across subpopulations.
